# Combinatorial strategies for cell transplantation in traumatic spinal cord injury

**DOI:** 10.3389/fnins.2024.1349446

**Published:** 2024-03-06

**Authors:** Vipin Jagrit, Jacob Koffler, Jennifer N. Dulin

**Affiliations:** ^1^Department of Biology, Texas A&M University, College Station, TX, United States; ^2^Department of Neurosciences, University of California, San Diego, La Jolla, CA, United States; ^3^Veterans Affairs Medical Center, San Diego, CA, United States; ^4^Texas A&M Institute for Neuroscience, Texas A&M University, College Station, TX, United States

**Keywords:** spinal cord injury, neural stem/progenitor cells, combinatorial therapies, neuromodulation, biomaterials

## Abstract

Spinal cord injury (SCI) substantially reduces the quality of life of affected individuals. Recovery of function is therefore a primary concern of the patient population and a primary goal for therapeutic interventions. Currently, even with growing numbers of clinical trials, there are still no effective treatments that can improve neurological outcomes after SCI. A large body of work has demonstrated that transplantation of neural stem/progenitor cells (NSPCs) can promote regeneration of the injured spinal cord by providing new neurons that can integrate into injured host neural circuitry. Despite these promising findings, the degree of functional recovery observed after NSPC transplantation remains modest. It is evident that treatment of such a complex injury cannot be addressed with a single therapeutic approach. In this mini-review, we discuss combinatorial strategies that can be used along with NSPC transplantation to promote spinal cord regeneration. We begin by introducing bioengineering and neuromodulatory approaches, and highlight promising work using these strategies in integration with NSPCs transplantation. The future of NSPC transplantation will likely include a multi-factorial approach, combining stem cells with biomaterials and/or neuromodulation as a promising treatment for SCI.

## Introduction

Spinal cord injury (SCI) has devastating consequences for the physical and social wellbeing of individuals. It affects more than 250,000 people each year worldwide, and has a significant impact on quality of life, life expectancy and financial wellbeing of people living with SCI (National Spinal Cord Injury Statistical Center, 2022). Traumatic SCI occurs by an external physical impact such as through a motor vehicle accident, fall, or sports-related injury. SCI typically results in permanent neurological deficits including impaired sensory, motor, and autonomic function. The primary mechanical injury is followed by a secondary injury cascade resulting in progressive cell death, inflammation, and scar formation, and the resulting cystic cavitation and reactive scar tissue creates a physical and chemical barrier for axonal regrowth (Thuret et al., 2006; Gomes et al., 2019). To date, despite significant progress in preclinical SCI research and a rapidly increasing number of clinical trials (Dietz et al., 2022), there are no proven-effective treatments that can improve neurological function after SCI.

“Neural stem/progenitor cells” is a catch-all term that refers to either neural stem cells, neural progenitor cells, or fetal-derived tissue that contains a mixture of both cell types. For the purposes of this review article, we will use neural stem/progenitor cells (NSPCs) as a general terminology, but will refer to the specific cell types used on a case-by-case basis as we discuss individual studies. In past decades, transplantation of NSPCs has shown great potential to reconstruct injured spinal cord tissue and promote functional recovery after SCI (Lane et al., 2017; Fischer et al., 2020). NSPCs are undifferentiated precursor cells of the central nervous system that have potential to differentiate into neurons, oligodendrocytes and astrocytes (McDonald et al., 1999; Cao et al., 2002; Han et al., 2002). NSPCs can be derived from human or rodent sources, either isolated from primary fetal or adult tissue or differentiated from pluripotent stem cells (Fischer et al., 2020). Transplanted NSPCs can also be differentiated to glial cells, providing scaffolds for axon regeneration as well as promoting remyelination and plasticity (Cummings et al., 2005, 2006; Wilcox et al., 2014; Nori et al., 2018). For the purposes of this review, we will focus on studies transplanting undifferentiated NSPCs in combination with different therapeutic strategies. These transplants possess beneficial properties capable of promoting repair and enhancing functional outcomes. The NSPC transplantation strategy is focused on promoting remyelination, modulating the immune response, providing neuroprotective benefits, and promoting neural relay formation (Fischer et al., 2020). NSPC grafts have been shown to promote axonal sprouting and regeneration by attenuating the growth inhibitory environment (Richardson et al., 1980; Bonner et al., 2011). Transplant-derived neurons can also anatomically and synaptically integrate into the host CNS, and even establish neuronal relays across the site of injury (White et al., 2010; Bonner et al., 2011; Lee et al., 2014; Spruance et al., 2018; Zholudeva et al., 2018). Additionally, multiple preclinical studies have reported improvement in voluntary motor function, respiratory function, sensory function, or bladder function following transplantation of NSPCs (Cummings et al., 2005; Lynskey et al., 2006; White et al., 2010; Lu et al., 2012; Lee et al., 2014; Dougherty et al., 2016; Fandel et al., 2016; Kadoya et al., 2016; Tashiro et al., 2016; Brock et al., 2018; Kumamaru et al., 2018; Rosenzweig et al., 2018; Zholudeva et al., 2018; Dugan et al., 2020; Kawai et al., 2021; Kitagawa et al., 2022). Together, these properties of neural transplants demonstrate their high potential to treat human SCI. Multiple clinical trials to evaluate safety and efficacy of NSPCs for SCI have been conducted (reviewed in Fischer et al., 2020). Recently, a first-in-human study evaluating safety of human induced pluripotent stem cell (iPSC)-derived NSPCs for human SCI has begun (Tsuji et al., 2019; Sugai et al., 2021).

Despite the promise of NSPC transplantation, there is a general consensus that there will be no single “magic bullet” therapy for SCI. Rather, combinatorial therapies may be more effective at achieving robust and reproducible functional improvements (Griffin and Bradke, 2020; Morse et al., 2021). In the context of NSPC transplantation, Pieczonka and Fehlings (2023) recently published a review of combinatorial techniques that can be used to direct transplanted NSPCs toward integration into targeted neural circuits, including molecular approaches to promote chemotaxis, task-specific rehabilitation, and galvanotaxis or magnet-based tools to promote graft migration. Here, we review previous and ongoing work to combine NSPC transplantation with two promising therapeutic approaches: biomaterials and neuromodulation.

## Combinatorial strategies integrating NSPCs with biomaterials

**FIGURE 1:**
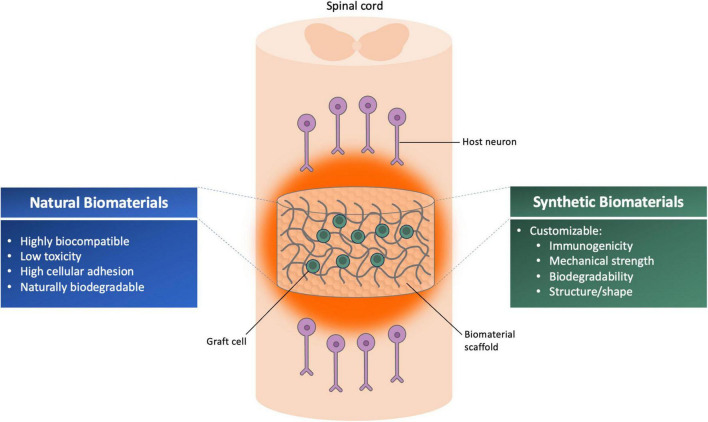
Illustration summarizing the advantages of different classes of biomaterial scaffolds for use with neural progenitor/cell transplantation therapy for spinal cord injury.

Biomaterials-based strategies are considered promising treatments for SCI that can stimulate axon regeneration, release growth-promoting factors, and promote functional recovery. Biomaterials are classified as degradable or non-degradable natural or synthetic polymers that can be implanted into the site of injury and used as bridges and/or delivery agents for cells, growth factors, or exosomes (Jeong et al., 2021). In recent years, there have been several published studies combining NSPCs with biomaterials. The main goal of this strategy is to deposit living cells on extracellular matrix that can provide a physical structure for cell growth and differentiation, guide growth and/or migration of grafted cells, and promote regeneration of host axons (Liu et al., 2019). Biomaterials can be used to make 3D scaffolds that promote cell survival, cellular interactions, proliferation, physical protection, and regeneration of cells while avoiding any adverse reactions to the organism (Marin et al., 2020; Feng et al., 2023). The idea of developing scaffolds is to mimic the extracellular environment of the spinal cord and reconstruct a favorable niche for SCI repair (Liu et al., 2019). At present, natural, synthetic and combined materials can be used to fabricate regenerative biomaterial scaffolds for SCI repair based on their different characteristics (Figure 1).

Natural materials have been widely used to develop various forms of scaffolds due to their advantageous properties such as excellent biocompatibility, biodegradability, and low toxicity (Libro et al., 2017). Natural polymers include nucleic acids, polysaccharides, proteins, lipids, and complex macromolecules such as proteoglycans. These natural polymers can form non-cytotoxic scaffolds either through self-assembly or using cross-linking techniques to encapsulate natural tissue properties (Joyce et al., 2021). Natural biomaterials mainly includes collagen, chitosan, hyaluronic acid, alginate, gelatin, agarose, and fibrin (Kim et al., 2000; Liu et al., 2019). Natural materials possess variety of advantages for use as biomaterial scaffolds for SCI repair but they also have inevitable disadvantages due to their intrinsic properties. For example, the mechanical properties of natural biomaterials cannot be easily manipulated as compared to synthetic biomaterials (Feng et al., 2023). For example, hyaluronic acid (HA) is a natural ECM component that is highly prevalent in the CNS, is known to promote regeneration, and is widely used in biomedical research (Jensen et al., 2020). However, HA may not necessarily be advantageous for severe SCI models; for instance, Koffler et al. (2019) demonstrated that a scaffold made of HA failed after transplantation of fetal rat spinal cord cells into an *in vivo* model of complete T3 spinal cord transection. The structure was not mechanically stable and collapsed by 4 weeks post-transplantation (Koffler et al., 2019).

Synthetic biomaterials are attractive to use due to their customizability. Their properties are often easier to tune than those of natural biomaterials; for example, porosity, stiffness, and degradation rates can be altered to match specific type of tissues (Feng et al., 2023). Some synthetic biomaterials possess strong mechanical properties, well-controlled biodegradability, low toxicity, low inflammatory response, customizable structure, and low immunogenicity (Subramanian et al., 2009). Widely used synthetic biomaterials include polyethylene glycol (PEG), polylactic acid, polylactic acid-hydroxyacetic acid copolymer, polyacrylamide, polyvinyl alcohol, polymethyl methacrylate, polyglycolic acid, and polycaprolactone. Among these polylactic acid, polyglycolic acid, and polycaprolactone are biodegradable synthetic polymers and are well known for their high mechanical strength, flexibility, and nontoxic degradability (Gunatillake and Adhikari, 2003; Shalumon et al., 2011). To improve efficacy of SCI repair, biomaterials can also be combined to form hybrid scaffolds that allow to incorporate the advantages of different single scaffolds and to overcome some disadvantages (Turnbull et al., 2018).

The elastic modulus as well as 3D architecture of biomaterial scaffolds are important considerations for experimental SCI studies. In one prominent study, Koffler et al. (2019) developed a method for 3D printing of biomimetic hydrogel scaffolds that mimic the architecture of a rodent spinal cord. Scaffolds were loaded with cells obtained from fetal (E14) rat spinal cords to support regeneration and form new neural relays across the site of injury. They observed axon outgrowth, graft survival, regeneration of host axons into scaffolds, and formation of synaptic connections between host axons and graft-derived neurons. In addition, the animals that received cell-loaded scaffolds recovered significantly greater locomotor function compared to the graft alone or scaffold alone groups (Koffler et al., 2019) (details of this and all subsequent studies highlighted in this article are described in detail in Table 1). Electrophysiological recordings before and after re-transection was performed suggest that establishment of *de novo* neural relays through the scaffolds are the underlying mechanism for this functional recovery (Koffler et al., 2019). Hence, this study demonstrated the importance of biomimetic materials to support directional growth of axons along conduits that resemble the architecture of the intact spinal cord. In another study, it was shown that a multichannel biodegradable polymer scaffold seeded with brain-derived fetal rat neural stem cells supported axonal regeneration after spinal cord transection in rats, with significantly greater host axon regeneration into neural stem cell (NSC)-containing scaffolds compared to scaffolds without cells. This study shows an effective use of multichannel biodegradable polymer scaffolds for quantitative analysis of axonal regeneration (Olson et al., 2009).

**TABLE 1:** Detailed descriptions of research studies highlighted in this review article.

Study	Cell type	Combinatorial therapy	Study details	Outcomes
**Biomaterials studies**				
Koffler et al., 2019	Neural progenitor cells (NPCs) isolated from E14 F344 rat spinal cord cells	3D-printed polyethylene glycol-gelatin methacrylate (PEG-GelMA) scaffolds	Scaffolds were loaded with cells and implanted into lesion sites of Fischer F344 rats with T3 complete spinal cord transection; outcomes were analyzed at 1 month and 6 months post-transplantation	NPC-loaded scaffolds featured significantly greater serotonergic axon innervation at 1 month and 6 months versus scaffolds alone; at 20 weeks, rats exhibited significantly greater locomotor function via BBB scoring at 5 months following treatment with NPC-loaded scaffolds (6.6 ± 0.5) compared to scaffolds alone (0.3 ± 0.2) or NPCx alone (1.6 ± 0.8); rats with NPC-loaded scaffolds exhibited significantly greater MEP amplitude (270 ± 5 μV) than rats with empty scaffolds (25.1 ± 5.7 μV)
Olson et al., 2009	NSCs isolated from E14.5 rat forebrain and cultured as neurospheres	Biodegradable 85:15 polylactic acid:polyglycolic acid (PLGA) scaffolds	Scaffolds were loaded with cells and implanted into lesion sites of Sprague Dawley rats with T8–9 complete spinal cord transection; outcomes were assessed at 1 month post-transplantation	Cell-loaded scaffolds were found to have significantly more neurofilament+ axons per transverse section than scaffolds without cells
Xu et al., 2021	NSPCs isolated from E14.5 Sprague Dawley rat hippocampus, cultured into spheroids, and dissociated	Decellularized tissue matrix hydrogels derived from spinal cord (DSCM-gel) or peripheral nerve (DNM-gel), and collagen type I hydrogels (COLI-gel)	NSPCs or spheroids were embedded in hydrogels and cultured *in vitro*; outcomes were assessed from 1 to 21 days in culture	NSPC spheroids cultured in DSCM-gel exhibited significantly greater cell proliferation and migration than those cultured in DNM-gel or COLI-gel; dissociated, differentiated NSPCs cultured in DSCM-gel contained significantly increased percentage of MAP2+ neurons and significantly decreased percentage of GFAP+ astrocytes compared to cells cultured in other hydrogel types; DSCM-gel promoted significantly more synaptogenesis *in vitro* compared to other gel types
Kourgiantaki et al., 2020	NSCs isolated from E13.5 C57BL/6 mouse cortex, cultured into neurospheres	Porous collagen-based scaffolds (PCSs)	NSC-seeded scaffolds or cell-free scaffolds were implanted immediately into lesion sites of mice with T13 dorsal column crush SCI; outcomes were assessed at 12 weeks post-injury	At 12 weeks post-SCI locomotor function, assessed by the horizontal ladder test, was significantly improved in NSC-scaffold group versus the injury-only group (8.4% ± 0.8% vs. 16.6% ± 1.6% error, respectively), but no difference between scaffold-only and injury only group; NSC-scaffold group exhibited increased axon density rostral to the lesion and decreased GFAP+ immunoreactivity around the lesion compared to injury-only
Geissler et al., 2018	NPCs isolated from E14–15 Sprague Dawley rat spinal cord, expanded in culture	Collagen-based hydrogels combined with laminin and hyaluronic acid (Col HA Lam)	Hydrogel was mixed with NPCs and transplanted into lesion sites of Sprague Dawley rats with C4 hemicontusion spinal cord injury at 1 week post-SCI; outcomes were assessed at 6 weeks post-transplantation	Rats receiving Col HA Lam-NPCs performed significantly better than rats receiving NPCs alone on the vibrissae-elicited forelimb placing test; rats receiving Col HA Lam-NPCs performed significantly better than rats receiving either media, Col HA Lam alone, or NPCs alone on the cylinder paw use preference test; the Col HA Lam-NPC group had significantly larger spared tissue area, and significantly decreased GFAP+ immunoreactivity, around lesions compared to media-only controls
Liu W. et al., 2023	Primary human NPCs isolated from human fetal spinal cord tissue at gestational 8 and 14 weeks, covalently modified with tetraacetylated *N*-azidoacetyl-*d*-mannosamine (Ac_4_ManNAz)	Longitudinally aligned collagen fibers (LACFs), modified with dibenzocyclooctyne groups	NPCs seeded onto LACFs were implanted into lesion sites of Sprague Dawley rats with T8–9 complete spinal cord transection immediately after injury; outcomes were assessed at 10–60 days post-transplantation	Numbers of transplanted cells retained at the lesion site were significantly higher, and fewer cells were apoptotic, in animals receiving cells that were covalently conjugated to LACFs versus cells that were noncovalently attached; animals receiving this treatment plus systemically administered liposomes exhibited a significantly higher BBB score (6 ± 0.75) and greater angle of inclined plane (38.3° ± 1.28°) than control groups
**Neuromodulation studies**				
Lai et al., 2023	Neural stem cells isolated from hippocampus of adult Sprague Dawley rats, cultured as neurospheres, transduced with AAV to express growth factors, seeded onto collagen sponge scaffolds, and cultured to produce spinal cord-like tissue (SCLT)	Tail nerve electrical stimulation (TNES) was performed at the base of the tail beginning on the 8th day post-surgery and administered for 20 min, five times a week for 8 weeks; stimulation strength of 20 mA, 4 kHz was utilized	SCLT was implanted into lesion sites of Sprague Dawley rats with T10 complete spinal cord transection immediately after injury; outcomes were assessed up to 8 weeks post-transplantation	Animals receiving TNES + SCLT implants recovered significantly greater locomotor function compared to animals receiving either treatment alone, as assessed by the BBB scale (difference of ∼1–5 points) as well as the inclined plane test; animals in the combined treatment group featured the highest density of neurofilament+ axons and MBP immunoreactivity caudal to the lesion site; animals in the combined treatment group had a greater degree of transsynaptic labeling across the site of injury compared to animals receiving SCLT alone
Liu W. et al., 2023	Neural stem cells isolated from E14 C57BL/6, EGFP, or PI3Kγ^–/–^ mouse cortex, cultured into neurospheres	Monolayer neural stem cells were subjected to direct physiological current electrical field (EF) at 100 mV/mm, 2 h/day, for 1–7 days	NSCs were transplanted into spinal cord at 1 mm rostral and caudal to the lesion sites of C57BL/6 mice with T8–9 contusion spinal cord injury at 1 week post-SCI; outcomes were assessed at 2, 4, and 8 weeks post-transplantation	EF-stimulated EGFP NSCs exhibited significantly greater survival than cells without stimulation at 14 days post-transplantation; EF-stimulated EGFP NSCs exhibited significantly greater length of neurite processes (14 and 28 days) and significantly more colocalization of processes with beta-III-tubulin (28 days) compared to cells without stimulation; mice receiving EF-stimulated NSCs recovered significantly greater locomotor function compared to all other treatment groups as assessed by the BBB scale (difference of ∼2.5 points)
Siddiqui et al., 2021	Schwann cells isolated from sciatic nerve of P2–5 Sprague Dawley pups, cultured, and transduced with retrovirus expressing GDNF	Cells were embedded into positively charged oligo [poly(ethylene glycol)fumarate] (OPF+) scaffolds, containing poly-lactic-co-glycolic acid (PLGA)-rapamycin microspheres; either a single channel isolated stimulator or an eight independent channel real-time programmable stimulator was used to deliver biphasic square wave epidural electrical stimulation (EES) to the S1 spinal cord segment (250 μs pulse width, 40 Hz 0.5–2.5 V)	Scaffolds containing cells were transplanted into the lesion sites of Sprague Dawley rats with T9 complete spinal cord transection immediately after injury; 1 week post-transplantation, animals received step training rehabilitation plus EES for 30 min/day, 3 days/week; outcomes were assessed up to 6 weeks post-transplantation	Animals that received combined EES + transplantation exhibited greater locomotor recovery than animals receiving either treatment alone, as assessed by the BBB scale (weeks 4 and 6 post-surgery) and kinematic analysis; animals with combined therapy contained greater densities of synaptic boutons colocalized with motor neurons and interneurons in the lumbosacral spinal cord versus animals with either treatment alone
Song et al., 2021	hNP1 human neural progenitor cells (hNPCs), cultured *in vitro*	Conductive nerve guides (CNGs) fabricated from polypyrrole (PPy) and attached to wires to allow for electrical stimulation	CNGs were seeded with alginate-encapsulated hNPCs and implanted into transected sciatic nerve; animals received electrical stimulation (ES) on day 1, 3, and 5 (40 V/m, 100 Hz, 1 h duration); outcomes were assessed up to 12 weeks post-transplantation	Animals that received combined CNGs + cells + ES exhibited significantly improved muscular gripping force compared to all other treatment groups (injury only, CNGs, CNGs + ES, CNGs + cells), significantly improved positional placement of hindpaw and extensor postural thrust versus injury-only animals; CNGs + cells + ES animals demonstrated significantly greater muscle mass compared to all other treatment groups; CNGs + cells + ES animals demonstrated significantly higher nerve conduction, amplitude of compound action potentials, and number of myelinated fibers in the nerve conduits compared to all other treatment groups

Note that the terminology referring to cell type (e.g., NSCs and NPCs) is consistent with the usage in each original article.

In recent years, decellularized tissue matrix (DTM) has garnered interest as a promising natural biomaterial for soft tissue repair or replacement therapeutics (Hussey et al., 2018). DTMs are prepared by either chemical or physical decellularization of mammalian tissues, removal of cellular antigens makes DTMs less immunogenic for host environment after implantation (Badylak, 2014). In one study, fetal rat hippocampus-derived NSPCs were embedded in DTM hydrogels (derived from either spinal cord or peripheral nerve) with low seeding density, and a large amount of cell proliferation was observed after 3 days of culture in DTM-gel (Xu et al., 2021). It was observed that NSPCs embedded in the hydrogel started spreading out and migrated farther into the DTM-gel within a 200-μm radius. The authors concluded that DTM-gel seeded with NSPCs facilitated stem cell proliferation and migration which might be because of its high porosity and tissue-specific components that provide a growth permissive environment. The authors also performed an *in vivo* study and reported that animals complete spinal cord transection had slightly improved hindlimb locomotor function if implanted with spinal cord-derived DTM compared to other hydrogels, but did not evaluate combined transplantation of NSPCs and hydrogels. This functional recovery was attributed by the authors to recruitment of endogenous NSPCs and enhancement of neurite sprouting toward the lesion gap (Xu et al., 2021). This study highlights the benefits of using DTMs; as these are decellularized form of mammalian tissue they are able to provide the native microenvironment and structure of the tissue. The DTM microenvironment may provide an ideal environment for cell transplantation and could reduce immunogenicity following transplantation into the injured spinal cord (Lee et al., 2018; Xu et al., 2021).

In another study, a porous collagen-based scaffold (PCS) was used to examine the proliferation, viability and neuronal differentiation of fetal mouse neural stem cells, as well as the therapeutic effect of NSCs on locomotor recovery following SCI (Kourgiantaki et al., 2020). The authors found that PCS-enabled delivery of NSCs at the SCI lesion site supported survival, axon ingrowth, and significantly reduced astrogliosis. These scaffolds seeded with NSCs supported significant locomotor functional recovery compared to injury-only controls, which the authors attribute to the ability of PCS to promote retention of seeded cells within the lesion site and graft integration with the host spinal cord (Kourgiantaki et al., 2020). Although some studies have used biomaterials for SCI treatment, clinical translation remains challenging as most of the biomaterials are not clinically approved. This study is remarkable in its demonstration of survival, differentiation of NSCs and functional recovery using a biomaterial that the authors indicate is similar to FDA-approved materials already utilized in the clinic for promoting regeneration skin and peripheral nerve wounds (Yannas et al., 1982, 1989; Soller et al., 2012).

Biomaterials may also provide a reliable and inexpensive way to control mechanical or chemical properties of the cellular environment, which is critical to direct cell behavior (Discher et al., 2009). Recent research has provided insight into the necessary chemical (Kang et al., 2007; Johnson et al., 2010; Fuhrmann et al., 2016) and mechanical properties (Brannvall et al., 2007; Rosenberg et al., 2008; Aurand et al., 2012) to direct differentiation of NSPCs toward neuron and astrocyte cell fate. For example, it was shown that a collagen, HA, and laminin-based hydrogel system could direct the differentiation of fetal rat NPCs into oligodendrocytes, potentially by mimicking ECM characteristics (Geissler et al., 2018). This study also reported significantly improved recovery of forelimb-dependent sensorimotor tasks in rats that received NPCs embedded in these hydrogels, versus controls. Although the authors did not explore the mechanism underlying functional recovery, they observed that animals receiving combined treatment exhibited significantly greater spinal cord tissue sparing and significantly decreased astroglial immunoreactivity compared to controls, suggesting a neuroprotective effect of treatment. This study shows that multiple-component biomaterials have potential to direct fates and therapeutic effects of co-transplanted cells (Geissler et al., 2018).

Recently, Liu W. et al. (2023) demonstrated that covalent conjugation between cells and biomaterials can be utilized to promote neural regeneration in a rat SCI model. Using a click chemistry approach, covalent conjugation of primary human spinal cord NPCs with collagen fibers promoted cell adhesion, prolonged cell retention on the scaffolds, and oriented axon outgrowth along the direction of fibers. Notably, differentiated cell types including astrocytes and Schwann cells were also utilized in this approach, and exhibited different effects on axon regeneration and vascularization. In an *in vivo* SCI experiment, the authors observed slight but significant improvements in locomotor function with combined treatment of NPC-seeded collagen fibers plus systemically administered liposomes, compared to other treatment groups, but mechanisms of recovery were not explored (Liu W. et al., 2023). These findings illustrate that chemical modification is a novel way to modulate interactions between transplanted cells and the biomaterial scaffold to promote cell retention and other desirable outcomes after SCI.

Natural and synthetic biomaterials have been widely used for clinical and preclinical investigations in biomedical research (Marin et al., 2020). Tissue engineering technology is a promising potential strategy to treat SCI through promoting regeneration of cells and tissue (Masaeli et al., 2019). However, there is still much work to be done toward optimizing biomaterials to promote robust and reproducible functional recovery following SCI. More research evidence are needed to optimize NSPC survival, regeneration and functional integration with host cells using biomaterials, especially in severe SCI models that do not support robust engraftment.

## Enhancing the therapeutic efficacy of NSPCs with neuromodulation

Neuromodulation therapy for SCI has developed rapidly in recent years. In fact, over 27% of SCI clinical trials initiated in 2021 employ some form of neuromodulation; more than any other intervention (Dietz et al., 2022). Neuromodulation involves alteration of nerve activity through targeted delivery of electrical or magnetic stimulation or ultrasound in order to alter neuronal activity (De Ridder et al., 2017; Vasanthan et al., 2019). There are invasive and noninvasive strategies for modulating neuronal activity. Invasive techniques include methods to stimulate the spinal cord, deep brain structures, or peripheral nerves using electrodes. Non-invasive techniques such as transcranial magnetic stimulation, transcranial direct current stimulation, epidural stimulation, and focused ultrasound utilize neuromodulatory devices that induce neuronal activity without penetrating the CNS. Neuromodulation is considered as one of the most promising treatment strategies for SCI because of the potential for electrically activating isolated neuronal circuits below the injury site which are intact but can no longer efficiently receives or transmit sensory information for processing (Squair et al., 2016). In recent years, several remarkable studies have reported significant neurological functional recovery in individuals with complete and chronic SCI and other neurodegenerative conditions (Harkema et al., 2011; Angeli et al., 2014; Rejc et al., 2015; Grahn et al., 2017; Gill et al., 2018; Darrow et al., 2019; Rowald et al., 2022; Lorach et al., 2023; Milekovic et al., 2023).

Various studies have also reported modest success in alleviating pain with epidural stimulation (Linderoth and Meyerson, 2010; Deer et al., 2014). The relatively noninvasive implantation of epidural electrodes led researchers to explore its other applications beyond pain relief (Kapural et al., 2016; Al-Kaisy et al., 2018; Deer et al., 2018). A case study including participants with chronic motor-complete SCI shows that epidural stimulation of lumbosacral spinal cord could enable them to make small leg movements with only stimulation, but when the stimulation was combined with physical training, participants was able to sustain contractions and generate force during leg flexion exercise (Angeli et al., 2014). In a follow up study, further improvements were reported with continued activity-based interventions without continuing stimulation (Rejc et al., 2017). These studies highlight the ability of electrical modulation to improve functional outcomes after spinal cord injury. Additionally, progressive improvements in the follow up study even after discontinuing stimulation might be because of neural adaptation during combined treatment with rehabilitation. However, it is important to keep in mind that enrollment in these clinical studies are limited to small numbers of patients; to get robust evidence of functional improvement, further studies including more SCI patients are necessary.

Combining NSPC transplantation with neuromodulatory approaches is a promising strategy for enhancing graft/host neural relay formation and neurological recovery following SCI (Courtine and Sofroniew, 2019; Zheng et al., 2020). To date, however, there have only been a handful of published studies combining these two approaches. Some potential clues about the potential effects of neuromodulation on transplanted NSPCs can be gleaned from studies of how *endogenous* NSPC behavior is altered by neuromodulation. TMS is used to noninvasively stimulate the nervous system via electromagnetic induction (Blackmore et al., 2019). In a recent study, it was found that low frequency repetitive TMS is effective in promoting endogenous brain NSC proliferation and neuronal differentiation (Abbasnia et al., 2015). Others have observed upregulation of neural stem cell growth promoting factors and neurotransmitters upon repetitive TMS (Feng et al., 2012). In support of that, Piacentini et al. (2008) showed that extremely low frequency electromagnetic fields exposure promotes NSC differentiation by upregulating Ca(v)1-channel expression and function. Together, these studies suggest that neuromodulation using electromagnetic fields could be a useful strategy to improve the injured microenvironment after SCI, through positively regulating cell proliferation and differentiation, release of neural stem cell growth promoting factors, and neurotransmitters.

Becker et al. (2010) showed that functional electrical stimulation (FES) from devices implanted adjacent to the peroneal nerve promoted progenitor proliferation following complete thoracic spinal cord transection. Rats received FES implants 3 weeks after T8/9 transection, and bromodeoxyuridine (BrdU) was injected 10 days later. The authors found that FES led to a near doubling in the birth and survival of BrdU^+^ cells that expressed nestin, a marker of multipotent neural progenitors, in the lumbar spinal cord (Becker et al., 2010). Another study examined the effects of electrical stimulation on spinal cord neurogenesis in rats after SCI (Bang et al., 2023). In this study, electrodes were used to deliver direct electrical stimulation to the spinal cord lesion site for 4 h/day from 2 to 6 weeks after T10 contusion SCI. Spinal cord neuronal differentiation was quantified at the study endpoint. Compared to animals that received SCI without stimulation, SCI + stimulation animals exhibited significantly greater numbers of neurons and nestin^+^ cells, suggesting increased neuronal differentiation; improved tissue sparing and locomotor recovery was also observed (Bang et al., 2023). Together, these studies indicate that either direct stimulation of the injured spinal cord or stimulation of the peripheral nerves can promote increased cell survival and neuronal differentiation following SCI, suggesting potential benefits for survival and/or proliferation of *engrafted* neural stem and progenitor cells.

To date, only a handful of studies have examined the effects of neuromodulation on NSPC engraftment and associated functional recovery after transplantation into sites of SCI. Recently, Lai et al. (2023) performed electrical stimulation of the tail nerve as a neuromodulatory approach to promote conduction of signals in neural stem cell-derived “spinal cord-like tissue” transplants in sites of complete spinal cord transection. Adult hippocampal rat NSCs expressing the neurotrophic factor NT-3 and receptor TrkC were cultured together with oligodendrocyte precursor cells expressing CNTF in collagen scaffolds to form the spinal cord-like tissue transplants, which were placed into sites of T10 transection SCI immediately following injury. Nerve stimulation was performed five times per week beginning at day 8 post-SCI and continuing once daily for 8 weeks. The authors found that the combination of nerve stimulation and transplantation resulted in the greatest locomotor functional recovery compared to SCI only, transplant only, and stimulation only groups, with animals in the combined treatment group achieving weight-supported stepping. These subjects also exhibited increased regeneration of neurofilament^+^ axons and myelination of regenerating axons, compared to other groups; additionally, transsynaptic tracing results also demonstrated a greater degree of neural connectivity across the lesion site in animals with combined treatment (Lai et al., 2023).

Liu W. et al. (2023) applied physiological electric field stimulation to NSCs *prior* to transplantation, in order to mimic the electrical potential that is present in the developing neural tube as a method to promote survival and differentiation. NSCs obtained from embryonic mouse brain were subjected to stimulation with a direct current electrical field, then transplanted into sites of spinal cord contusion injury. Following transplantation, the authors reported a significantly higher survival rate in stimulated NSCs versus unstimulated NSCs (40% ± 6.6% versus 21.9% ± 9.1%, respectively), as well as increased neuronal differentiation and neurite outgrowth from stimulated transplants. The PI3K/Akt/GSK-3β/β-catenin signaling cascade was shown to be required for the effects of electrical field stimulation on boosting neuronal differentiation. Mice receiving transplants of stimulated NSCs performed better on locomotor functional assessments versus mice that received unstimulated transplants, presumably due to enhanced graft survival following stimulation (Liu W. et al., 2023).

In another study, a neuroregenerative scaffold in combination with epidural electrical stimulation (EES) was found to promote functional recovery in rats with complete spinal cord transection (Siddiqui et al., 2021). Siddiqui et al. (2021) used hydrogel scaffolds composed of positively charged oligo-[poly(ethylene glycol)fumarate] loaded with neurotrophic factor (GDNF)-secreting rat Schwann cells and rapamycin microspheres. Scaffolds were seeded with GDNF-expressing Schwann cells and rapamycin microspheres, and transplanted into sites of complete T9 transection. Animals received EES via implanted electrodes over the sacral (S1) spinal cord to enable motor training on a treadmill, such that stimulation enabled stepping (EES-facilitated training). Animals received manual bipedal step training rehabilitation 3 days/week for 6 weeks after SCI under the influence of EES. The authors found that combined treatment with scaffolds and EES-enabled stepping led to significantly greater locomotor functional improvement as compared to individual treatment or control groups by 6 weeks post-injury (Siddiqui et al., 2021). This recovery could be due to effects of treatment on plasticity, as animals with combined therapy contained greater densities of synaptic boutons colocalized with motor neurons and interneurons in the lumbosacral spinal cord versus animals with either treatment alone. This study shows the use combinational approach of cell-containing scaffold with electrical stimulation to demonstrate that regenerated axons through the length of the scaffold can reorganize the neural circuitry around SCI and improve motor performance. More studies like this are needed to understand the mechanism behind the recovery and therapeutic effects of these combined interventions and to maximize functional restoration after SCI.

Finally, electrically conductive biomaterials can be used in conjunction with NSPCs as a method to promote neuromodulation of grafted cells. Serafin et al. (2023) demonstrated recently a combinatorial approach using natural biomaterials (hyaluronic acid and gelatin) in combination with polypyrrole (PPy) nanoparticles to create conductive scaffolds. PPy is the most commonly studied conductive biomaterial (George et al., 2017; Oh et al., 2018). The authors found that mesenchymal stem cells seeded on these scaffolds attached well and proliferated *in vitro* (Serafin et al., 2023). The same group used novel poly(3,4-ethylenedioxythiophene) nanoparticles to increase the conductivity of hydrogel scaffolds as a therapeutic approach for spinal cord injury (Serafin et al., 2022). In this *in vivo* study, using a model of T3 complete spinal cord transection in rats, the authors showed high conductivity, and reduced gliosis and inflammatory response in the animals receiving scaffolds. In another study, conductive polymer made of PPy allowed for electrical stimulation of human neural progenitor cells (hNPCs) implanted within the biomaterial. Song et al. (2021) applied electrical stimulation in a rat sciatic nerve transection model and found that electrical stimulation of the nerve combined with human neural progenitor cell-laden scaffolds significantly promoted nerve regeneration, nerve conduction, myelination, and recovery of nerve function in rats after sciatic nerve transection. The dramatic improvement in functional recovery observed in this study is attributed by the authors to increased myelination and a greater degree of neurotrophic factor release. These results highlights the positive effect of combinational approaches using conductive materials and electrical stimulation of transplanted stem/progenitor cells to enhance therapeutic potential of stem cell therapy.

Neuromodulation is a rapidly growing field itself with great potential for treatment of SCI, evident by a growing number of clinical trials and a great deal of experimental evidence of functional recovery (Harkema et al., 2011; Ho et al., 2014; Ajiboye et al., 2017). Hence, combinatorial strategies incorporating regenerative therapies such as NSPC transplantation with neuromodulation are likely to lead to the next generation of SCI therapies. As discussed in this review, there is emerging evidence to suggest that neuromodulation intervention can augment the effects of regenerative therapies after SCI. However, much more work is needed to evaluate the therapeutic efficacy of combined cell transplantation and electrical or magnetic stimulation strategies, and to determine the mechanisms by which these therapies alone or in combination can promote functional recovery.

## Discussion

Neural stem/progenitor cell transplantation therapies, use of biomaterial scaffolds, and neuromodulation techniques has been extensively used in efforts of improving functional outcomes after SCI. Modest gain in functional outcomes has been observed following treatment with these therapies, but not complete recovery, as per the complex nature of SCI the pathology cannot be fully addressed by a single therapeutic approach. The lack of success may be addressed by a combinatorial or multi-disciplinary approach, where multiple interventions can be used as an integrated approach for SCI. Thus, a critical point for future research is to optimize the degree of functional recovery that can be achieved after SCI.

In this review we highlight and promote the use of NSPC transplantation therapy combined with biomaterial scaffold and neuromodulation techniques to improve anatomical and functional recovery after SCI. Nevertheless, work needs to be done to find out whether any of these therapies when combined with NSPC can produce cumulative improvements after human SCI. To validate the efficacy of combined therapies, preclinical studies should be reproduced by multiple laboratories to increase confidence in positive results.

## Author contributions

VJ: Writing – original draft, Writing – review & editing. JK: Funding acquisition, Writing – original draft, Writing – review & editing. JD: Funding acquisition, Writing – original draft, Writing – review & editing.
